# Endometrium development patterns and BMI groups among *in vitro* fertilization patients; prognostic aspects

**DOI:** 10.3389/fendo.2024.1379109

**Published:** 2024-04-26

**Authors:** Viktor Vedelek, Petra Bicskei, Mariann Tábi, Noémi Lajkó, Csaba Ékes, Kristóf Bereczki, Zsófia Meixner-Csáti, Rita Sinka, Anna Vágvölgyi, János Zádori

**Affiliations:** ^1^ Department of Genetics, Faculty of Science and Informatics, University of Szeged, Szeged, Hungary; ^2^ Institute of Reproductive Medicine, Albert Szent-Györgyi Medical Centre, University of Szeged, Szeged, Hungary; ^3^ Department of Medicine, Albert Szent-Györgyi Medical School, University of Szeged, Szeged, Hungary

**Keywords:** *in vitro* fertilization, clinical pregnancy rate, endometrium thickness, obesity, body mass index

## Abstract

**Introduction:**

The impact of the obesity pandemic on female reproductive capability is a factor that needs to be investigated. In addition, the link between endometrial thickness and *in vitro* fertilization (IVF) outcomes is contentious.

**Goal:**

Our goal was to analyze the association among endometrium development, hormone levels, embryo quality, clinical pregnancy, anamnestic parameters, and body mass index (BMI) in women receiving IVF treatment.

**Patients and methods:**

537 participants undergoing IVF/ICSI cycles with successful oocyte retrieval were enrolled. Subjects were divided into four BMI based groups: underweight (UW; n=32), normal weight (NW; n=324), overweight (OW; n= 115), obesity (OB; n=66). Anthropometric and anamnestic parameters, characteristics of stimulation, endometrial thickness on the day of hCG injection, at puncture, at embryo transfer, FSH, LH, AMH, partner’s age and the semen analysis indicators, embryo quality, clinical pregnancy, were recorded and analyzed. Support Vector Machine (SVM) was built to predict potential pregnancies based on medical data using 22 dimensions.

**Results:**

In accordance with BMI categories, when examining pregnant/non-pregnant division, the average age of pregnant women was significantly lower in the UW (30.9 ± 4.48 vs. 35.3 ± 5.49 years, p=0.022), NW (34.2 ± 4.25 vs. 36.3 ± 4.84 years, p<0.001), and OW (33.8 ± 4.89 vs. 36.3 ± 5.31 years, p=0.009) groups. Considering FSH, LH, and AMH levels in each BMI category, a statistically significant difference was observed only in the NW category FSH was significantly lower (7.8 ± 2.99 vs. 8.6 ± 3.50 IU/L, p=0.032) and AMH (2.87 ± 2.40 vs. 2.28 ± 2.01 pmol/L, p=0.021) was higher in pregnant women. There were no further statistically significant differences observed between the pregnant and non-pregnant groups across any BMI categories, especially concerning endometrial development. Surprisingly, BMI and weight correlated negatively with FSH (r=-0.252, p<0.001; r=-0.206, p<0.001, respectively) and LH (r= -0.213, p<0.001; r= -0.195, p<0.001) in the whole population. SVM model average accuracy on predictions was 61.71%.

**Discussion:**

A convincing correlation between endometrial thickness development and patients’ BMI could not be substantiated. However, FSH and LH levels exhibited a surprising decreasing trend with increasing BMI, supporting the evolutionary selective role of nutritional status. Our SVM model outperforms previous models; however, to confidently predict the outcome of embryo transfer, further optimization is necessary.

## Introduction

The global prevalence of obesity has surged in recent decades, reaching pandemic proportions. Hungary is ranked by the latest data of World Obesity Global Obesity Observatory (1) among the countries with elevated rates of overweight (24^th^ place in the global ranking, 29.12% affected of the female population) and obesity (91^st^ place in the global ranking, 25.64% affected of the female population). As a major problem of public health, beyond the consequences of obesity, such as cardiovascular and metabolic diseases, several cancers, obstructive sleep apnea, dementia, declined life quality and expectancy. Obesity also contributes to reproductive dysfunction. Obesity is associated with increased conception time (2, 3) and decreased fecundity (2, 4–8). Infertility affects approximately 15–20% of couples aspiring to have children in Hungary, and according to the data of Ministry of Human Capacities (9) 1.5-2% of children are owed to assisted reproductive technology (ART). According to the recommendations of National Institute for Health and Care Excellence (NICE) women should be advised that it is preferable for their BMI to fall within the range of 19–30 kg/m^2^ before initiating assisted reproduction, as a BMI outside this range is likely to decrease the success rate of ART procedures (10). In the largest cohort study conducted so far (11), increasing body mass index (BMI) was associated with decreased pregnancy and live birth and increased pregnancy loss following euploid frozen embryo transfer across the entire cohort and specifically among patients diagnosed solely with polycystic ovarian syndrome (PCOS). Nevertheless, these findings were less pronounced among patients with a sole diagnosis of male factor infertility, implying that the trend may be attributed to associate female infertility diagnoses rather than BMI alone.

Once the conceptus arrives, thrivingly breaches the uterine mucosa and later embeds the endometrial stroma, the enigmatical early period of implantation starts. Endometrium appears as a dynamic and reactive tissue. Human reproduction is characterized by a degree of embryo wastage, which is dominantly ascribed to a high prevalence of embryo aneuploidy. Key to this is the human decidualized endometrium as a sensor of embryo quality (12). The decidualization process includes two components: the differentiation of endometrial stromal cells to decidual stromal cells, as well as the recruitment and education of decidual immune cells. At the maternal-fetal interface, stromal cells undergo morphological and phenotypic changes and interact with trophoblasts and decidual immune cells to provide an appropriate decidual bed and tolerogenic immune environment to maintain the survival of the semiallogeneic fetus without causing immunological rejection (13). Synchrony between progesterone-driven endometrial receptivity and the arrival of an euploid blastocyst is essential for embryo implantation. Soon after embryo attachment and the early stages of implantation, further invasion into the maternal compartment requires a functional decidua (14). However, even in the case of transferring high reproductive competence euploid blastocysts the implantation rate may exceed 50%, the prognosis of a successful pregnancy is highly determined by the endometrium development in the stimulation period and after starting progesterone support following human chorionic gonadotropin (hCG) triggering and follicular puncture. The prognostic value of the endometrium development is ascertained. Sonographic parameters, such as endometrial thickness, and endometrial pattern are intensely used for monitoring the stimulation and predicting the outcome. Although numerous studies focusing on the relationship between endometrial thickness and *in vitro* fertilization (IVF) outcomes have been conducted, no consensus has been reached and the association remains controversial (15). Confounding factors associated with maternal age, such as variability in ovulation stimulation protocols, the number of transferred embryos, and interobserver divergence, as well as specific patient groups like those with PCOS or obesity, could be potential reasons for this inconsistency.

## Goals

Analysis of the relationship among the maternal BMI, the clinical pregnancy rate and the endometrial thickness consequent measured by the same IVF specialist as only one observer to avoid observer bias. In addition, a Support Vector Machine (SVM) model was built to predict potential pregnancies based on medical data using 22 dimensions.

## Patients and methods

### Study population

A retrospective, single-center cohort study was conducted in the Institute of Reproductive Medicine, University of Szeged in cooperation with the Endocrinology and Diabetology Outpatient Clinic at the Department of Medicine, Albert Szent-Györgyi Medical School, University of Szeged. The data collection was performed from January 1, 2020, to March 31, 2023 among women treated for IVF with or without intracytoplasmic sperm injection (ICSI) with successful oocyte retrieval, who were followed and monitored by the same experienced physician. The following parameters were recorded by all subjects: age (year), weight (kg), height (cm), body mass index (BMI; kg/m^2^), endometrial thickness on the day of triggering hCG injection (ENDOV; mm), endometrial thickness at follicular puncture (ENDPU; mm), endometrial thickness at embryo transfer (ENDET; mm), follicle-stimulating hormone (FSH; IU/L), luteinizing hormone (LH; IU/L), and anti-Müllerian hormone (AMH; pmol/L). The anamnestic duration of infertility (years), the fact of Fallopian tube obstruction, unsuccessful intrauterine insemination (IUI), duration of stimulation (days), number of discontinued cycles, number of follicles, total number of transferred embryos, and the embryo score of the transferred best embryo were also documented and collected. On the male side, the partner’s age (year) and the indicators of semen analysis were recorded, such as sperm concentration (x 10^6^/ml), sperm motility (%), and morphology (% of sperm with normal shape and structure). As an outcome, the occurrence or absence of clinical pregnancy was recorded. Clinical pregnancy was defined at 7 weeks gestational age, as recommended by the International Committee for Monitoring Assisted Reproductive Technology (16). This is defined by the visualization of one or more gestational sacs (including ectopic pregnancies) using transvaginal ultrasound. Biochemical pregnancy, clinical miscarriage, and ectopic pregnancy (16) were recorded separately, and patients with these outcomes were not included in the pregnant group.

Inclusion required the complete availability of the above-mentioned parameters. During the aforementioned time interval, 832 women underwent *in vitro* fertilization and embryo transfer (IVF-ET) treatment at the Institute of Reproductive Medicine, University of Szeged. Out of the study population, complete data were available for 545 patients. Eight patients were excluded, one due to an ectopic pregnancy and seven due to biochemical pregnancies, as they could not be classified into either the non-pregnant or clinical pregnancy subcategories. Their limited occurrence precluded their inclusion in the statistical analysis. The data of the remaining 537 patients was subsequently subjected to analysis.

Subjects were primarily divided according to the World Health Organization (WHO) classification into the six following groups: underweight (UW; BMI<18.5 kg/m^2^; n=32), normal weight (NW; BMI: 18.5–24.9 kg/m^2^; n=324), overweight (OW; BMI: 25–29.9 kg/m^2^; n= 115), class I obesity (BMI: 30–34.9 kg/m^2^; n=54), class II obesity (BMI: 35–39.9 kg/m^2^; n=11) and class III obesity (BMI ≥ 40 kg/m^2^; n=1). Class I, II and III obesity were gathered due to the small low case numbers; it is referred in subsequent sections as group obesity (OB; BMI ≥ 30 kg/m^2^; n=66).

### Methods

Adjusted to the women’s ovarian function, sex hormone profile, age, and body weight, patient tailored flexible gonadotropin-releasing hormone (GnRH) antagonist protocols were implemented for ovarian stimulation. Cycle selection was performed on day 2/3. This initial visit included ultrasound control (Samsung Medison HS50; endocavitary probe: EVN4-9, 4-9 MHz), antral follicle count estimation, FSH, LH, prolactin and thyroid-stimulating hormone (TSH) evaluation. FSH treatment started on the 2^nd^–3^rd^ day of menstruation, and it was adjusted according to patient response, who was monitored by serial transvaginal sonography and estrogen (E2) level from day five and every 2–3 days on. Once the leading follicles got to the diameter of 14 mm, 0.25 mg of GnRH antagonist was given daily until the day of trigger. Triggering was performed by hCG, when the patient had at least three follicles that were ≥18 mm. 36 hours after the hCG (Ovitrelle^®^, Merck) triggering, the oocytes were removed by transvaginal oocyte retrieval. Laboratory and transfer procedures were similar in all cycles and followed general laboratory protocols. The follicular fluid was collected in preheated round bottom 14 ml tubes (Thermo Fisher Scientific, Denmark) and held in a heating block calibrated at 37°C. The oocyte search was performed under laminar flow using 90 mm petri dishes (Thermo Fisher Scientific, Denmark). The cumulus-oocyte complexes were collected in Nunc IVF Center Well Dish (Thermo Fisher Scientific, Denmark) in G-MOPS PLUS (Vitrolife, Sweden) medium. Subsequently, the complexes were placed in 5-well dishes (Vitrolife, Sweden) in G-IVF (Vitrolife, Sweden) under oil overlay (Hypure Heavy Ovoil, Kitazato, Japan) in incubator with conditions set at 37°C, 6% CO_2_ and 5% O_2_ (K-Systems G210 InviCell, CooperSurgical, Denmark and PLANER Benchtop Incubator BT37, CooperSurgical, Denmark). Fertilization was performed 2-4 hours after oocyte retrieval, either through conventional IVF or through ICSI, depending predominantly on the semen parameters. For conventional IVF, only fresh semen with normal quality (normozoospermia) was applied to fertilize cumulus–oocyte complexes distributed in the wells of 5-well dishes in G-IVF media under oil overlay. Oocytes were denuded using hyaluronidase enzyme (80 IU/ml; SynVitro Hyadase, Origio, Denmark) for ICSI. Following fertilization, they were incubated in G1 and G2 (Vitrolife, Sweden) media in a 5-well dishes (Vitrolife, Sweden) in an incubator with conditions of 6% CO_2_, 5% O_2_ and 37°C (K-Systems G210 InviCell, CooperSurgical, Denmark, PLANER Benchtop Incubator BT37, CooperSurgical, Denmark). At 17–20 hours after successful insemination, zygotes were examined for the presence of two pronuclei including the paternal and the maternal pronucleus. If more than two pronuclei were observed in a zygote, it was classified as an abnormal zygote and discarded. For transfer, the best quality embryos at the cleavage- or blastocyst-stage were selected. However, only blastocyst-stage embryos were chosen for freezing on culturing day 5 or 6. Embryos were qualified based on number of blastomeres and percentage of fragmentation (17). Before embryo transfer, blastocysts were placed in EmbryoGlue (Vitrolife, Sweden) for 20-40 minutes. The embryos were transferred intrauterine with the Wallace Embryo Replacement Catheter (soft, 18 cm, CooperSurgical, USA). Two-dimensional transvaginal ultrasonography (Samsung Medison HS50; endocavitary probe: EVN4-9, 4-9 MHz) was utilized for follicular punctures and embryo transfers. The endometrial thickness was also measured by the same ultrasound device on the day of triggering hCG injection (ENDOV; mm), at puncture (ENDPU; mm), and at the time of embryo transfer (ENDET; mm), always by the same examiner. Endometrial measurements were taken from the outer edge of the endometrial-myometrial interface to the outer edge in the widest part of the endometrium (18). An individual evaluation system was devised to normalize differences based on the development level and quality of embryos on different transfer days. Utilizing a distinct embryo point score for statistical analysis, the corresponding details are summarized in **Table 1**.

**Table 1:** Embryo scoring system.

Day of culturing/Quality of embryo	Excellent quality (3 points)	Good quality (2 points)	Poor quality (1 point)
**Day 3**	8A1, 8B1, 10A1, 10B1	6A1, 6B1, 8A2, 6A2, 10A2, 12A1	8B2, 10B2, 6B2, 4A1, 2A1 or worse quality
**Day 4**	compact morula, early blastocyst	10A1, 10B1, 10A2, 12A1, early morula	8A1, 10B2 or worse quality
**Day 5**	1AA, 2AA, 3AA, 4AA, 5AA, 6AA	1BB, 1BA, 1AB, 2BB, 2AB, 2BA, 3BB, 3BA, 3AB, 4BB, 4BA, 4AB, early blastocyst, compact morula	1CC, 1BC, 1CB, 2CC, 2CB, 2BC, 3CC, 3CB, 3BC, 4CC, 4BC, 4CB, early morula, 12A1 or worse quality

Embryo scoring system combined and modified after Gardner et al. (19) and Irani et al. (20); expansion status is described on a numerical scale from 1-6, while trophectoderm and inner cell mass are both categorized using letter A-B-C.

The quality of embryos transferred is a key factor determining the success or failure of IVF treatment cycles. The detailed morphological embryo grading system by Gardner et al. (19) reliably represents the embryo quality and provides standardization in the assessment. The integration of embryo quality into statistical analysis is a major requirement for evaluating the outcome of IVF treatment cycles. To advance both research and clinical processes, the expansion status is described on a numerical scale from 1-6, while trophectoderm and inner cell mass are both categorized using letter A-B-C. The combinations of these variables result in 54 possible categorical embryo grades. To simplify the analysis and reduce the number of categorical variables, the embryo grade subgroups were clustered into three groups. Clustering a large number of embryo grades into classifications such as ‘excellent, good, or poor’ may lead to some data loss, but it enchases the interpretability of the analysis (20).

### Statistical analysis

For general data handling, we utilized Microsoft Excel. For data analyses and discovery, the Jupyter notebook v. 6.3.0 was used. Python 3.6, numpy, pandas, scipy and sklearn libraries for data management and statistical analyses were applied. Welsch’s two-tailed significance test was used to determine significance. Values of p<0.05 were considered significant. The Pearson coefficient was calculated to determine correlation, and the corresponding two-sided p-values were calculated using the scipy.stats.pearsonr function. Cohen’s effect size (d) calculation was implemented in Python 3.6. Statistical power calculations were conducted using TTestIndPower function from statsmodels library.

Scikit-learn implementation was employed for k-means clustering and stratified K-fold cross-validation. To predict potential pregnancies based on medical data, Support Vector Machine models were also constructed using the scikit-learn library. For visualization, Python 3.6 matplotlib and seaborn libraries were utilized.

Enrichment was calculated as follows: (n_out_/n_count_)/(N_out_/N_count_), where n_out_ represents the number of investigated outcomes in the examined group, n_count_ represents the number of elements in the group, N_out_ represents the total number of investigated outcomes, and N_count_ represents the total number of elements.

## Results

### BMI categories and clinical data of the whole population

The subjects of the study were divided into four categories (UW, NW, OW, OB) based on BMI indices, as outlined in the methods section. **Table 2** contains the basic statistical characterization of the established groups. For further investigation, significant differences in clinical data between categories were determined (**Supplementary Table 1**).

**Table 2:** Relevant clinical data in the study groups.

Clinical Data	UW (n=32)	NW (n=324)	OW (n=115)	OB (n=66)
**Age (year)**	33.6 ± 5.49	35.5 ± 4.72	35.3 ± 5.27	35.1 ± 5.50
**Weight (kg)**	49.5 ± 5.74	60.0 ± 6.75	73.5 ± 6.86	90.0 ± 9.58
**Height (cm)**	167.7 ± 5.82	166.2 ± 6.13	165.1 ± 6.58	165.5 ± 6.53
**BMI (kg/m^2^)**	17.5 ± 1.30	21.7 ± 1.80	26.9 ± 1.41	32.8 ± 2.40
**Number of pregnant patients (n; %)**	12 (37)	129 (40)	48 (42)	31 (47)
**Number of non-pregnant patients (n; %)**	20 (63)	195 (60)	67 (58)	35 (53)
**Duration of infertility (years)**	4.3 ± 3.65	4.4 ± 2.70	4.8 ± 3.90	6.1 ± 3.57
**Number of Fallopian tube obstruction (n; %)**	4 (13)	70 (22)	34 (30)	15 (23)
**Number of unsuccessful IUI (n; %)**	8 (25)	87 (27)	19 (17)	8 (12)
**FSH** (**IU/L)**	8.9 ± 2.73	8.3 ± 3.33	7.4 ± 2.81	5.8 ± 1.44
**LH** (**IU/L)**	6.3 ± 3.52	6.5 ± 2.43	5.7 ± 3.01	5.0 ± 2.71
**AMH** (**pmol/L)**	2.9 ± 3.57	2.5 ± 2.19	2.8 ± 2.84	3.2 ± 2.45
**Duration of stimulation (day)**	9.8 ± 1.39	10.2 ± 1.85	10.6 ± 1.71	11.2 ± 2.17
**Number of discontinued cycles**	0.16 ± 0.37	0.16 ± 0.50	0.13 ± 0.39	0.11 ± 0.36
**Number of follicles**	6.5 ± 2.69	6.8 ± 2.95	6.8 ± 3.08	7.1 ± 2.72
**Total number of transferred embryos**	1.5 ± 0.62	1.8 ± 0.59	1.8 ± 0.61	1.7 ± 0.59
**Embryo score of the transferred best embryo**	2.3 ± 0.69	2.5 ± 0.66	2.6 ± 0.65	2.3 ± 0.75
**ENDOV (mm)**	9.5 ± 2.36	10.0 ± 1.82	9.7 ± 1.98	10.6 ± 1.84
**ENDPU (mm)**	10.2 ± 2.41	10.5 ± 2.02	10.2 ± 1.86	11.1 ± 2.03
**ENDET (mm)**	11.7 ± 2.27	11.8 ± 2.23	11.8 ± 2.38	12.2 ± 2.22
**Paternal age (year)**	38.1 ± 5.76	38.7 ± 6.14	38.7 ± 6.05	38.0 ± 5.71
**Sperm concentration (x 10^6^/ml)**	49.8 ± 40.26	54.4 ± 45.98	42.1 ± 43.15	46.3 ± 39.44
**Sperm motility (%)**	46.7 ± 22.25	44.5 ± 18.53	41.2 ± 18.11	43.3 ± 17.58
**Teratozoospermia (n; %)**	14 (44)	101 (31)	47 (41)	22 (33)
**Normal sperm morphology > 4% (n; %)**	18 (56)	223 (69)	68 (59)	44 (67)

The data are presented as mean ± SD. IUI, intrauterine insemination; FSH, follicle-stimulating hormone; LH, luteinizing hormone; AMH, anti-Müllerian hormone; ENDOV, endometrial thickness on the day of triggering hCG injection; ENDPU, endometrial thickness on the day of puncture; ENDET, endometrial thickness at the time of embryo transfer.

**Figure 1:**
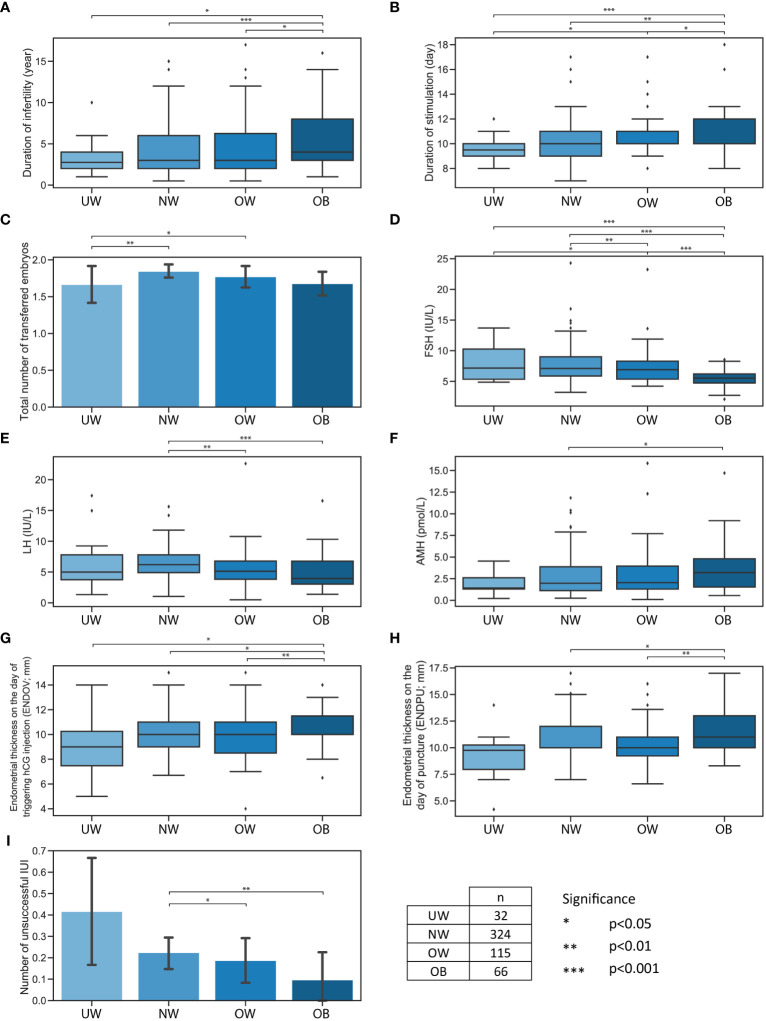
Significant differences between four BMI categories. Significant differences between four BMI categories: UW, NW, OW, OB. **(A)** Boxplot represents the duration of infertility. **(B)** Boxplot represents the duration of stimulation. **(C)** Bar plot represents the total number of transferred embryos. **(D)** Boxplot represents FSH levels. **(E)** Boxplot represents LH levels. **(F)** Boxplot represents AMH levels. **(G)** Boxplot represents endometrial thickness on the day of triggering hCG injection. **(H)** Boxplot represents endometrial thickness on the day of puncture. **(I)** Bar plot represents the number of unsuccessful IUI. Bar plot error bars indicate 95% confidence intervals. Significance boundaries and sample numbers are available at the bottom right corner.

The duration of infertility was significantly longer in the OB category compared to NW (p<0.001; ***), OW (p=0.020; *) and to UW (p=0.025; *) (**Figure 1A**). The duration of the stimulation in the OB group was significantly longer compared to the UW (p<0.001; ***), NW (p=0.001; **) and OW (p=0.042; *) categories. Taking this into consideration, it is worth noting that the duration of stimulation exhibited a significant increase in the OW compared to the UW category (p=0.001; *) (**Figure 1B**). The total number of transferred embryos were significantly higher in the NW (p=0.009; **) and OW (p=0.011; *) categories compared to the UW category (**Figure 1C**). With the increase in BMI, there was a significant decrease in serum FSH and LH levels. FSH was significantly lower in the OW (p=0.012; *) and in the OB (p<0.001; ***) categories compared to the UW category. The identical trend was apparent in OW (p=0.008; **) and in the OB (p<0.001; ***) categories, as FSH was also significantly lower compared to the NW category. Additionally, FSH in OB category was significantly lower (p<0.001; ***) in comparison to OW category (**Figure 1D**). LH was significantly lower in OW (p=0.008; **) and OB (p<0.001; ***) categories compared to the NW category (**Figure 1E**). AMH was slightly but significantly increased only in the OB category compared to the NW group (p=0.045; *) (**Figure 1F**). According to endometrial thickness, only the ENDOV was significantly thicker in the OB group in comparison to the UW group (p=0.027; *), to the NW group (p=0.035; *); and to the OW group (p=0.004; **) (**Figure 1G**). Additionally, ENDPU appeared statistically thicker in the OB category compared to NW (p=0.038; *) and in category OB compared to OW (p=0.003; **) (**Figure 1H**). The number of unsuccessful IUI attempts was significantly lower in the OB (p=0.02; **) and OW (p=0.016; *) categories compared to the NW category (**Figure 1I**).

### BMI categories and Pregnant/Non-pregnant subgroups

**Figure 2:**
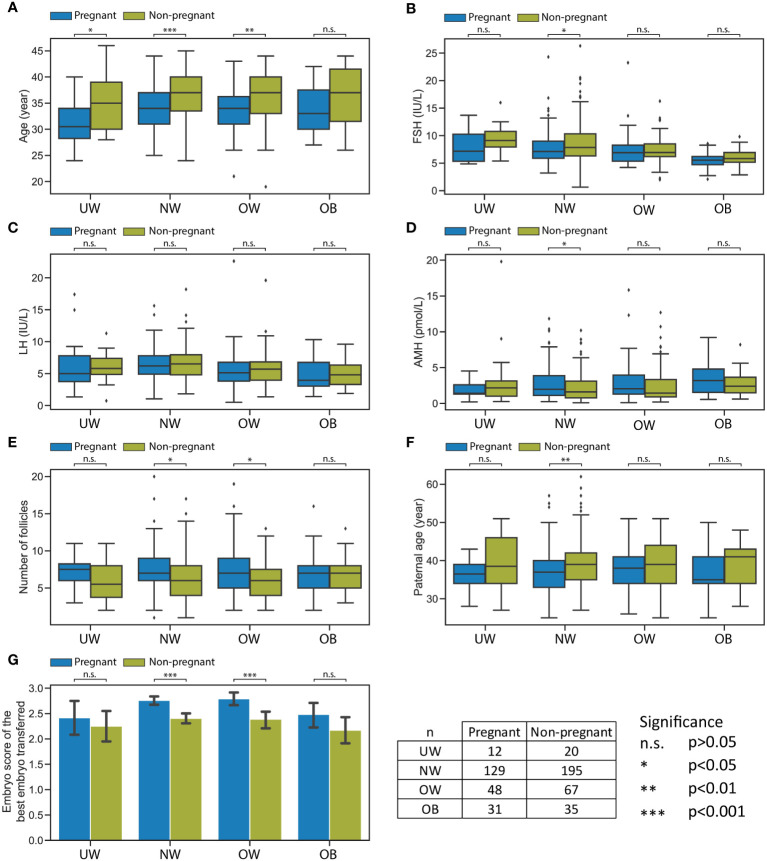
Significant differences between pregnant and non-pregnant subgroups within the four BMI categories. Significant differences between pregnant and non-pregnant subgroups within the four BMI categories: UW, NW, OW, OB. **(A)** Boxplot represents age. **(B)** Boxplot represents FSH levels. **(C)** Boxplot represents LH levels. **(D)** Boxplot represents AMH levels. **(E)** Boxplot represents the number of follicles. **(F)** Boxplot represents paternal age. **(G)** Bar plot represents the embryo score of the best embryo transferred. Bar plot error bars indicate 95% confidence intervals. Significance boundaries and sample numbers are available at the bottom right corner.

In accordance with BMI categories, when examining the pregnant/non-pregnant division within the patient group, the following significant differences were confirmed (**Supplementary Table 1**). The average age of women who successfully conceived was significantly lower in the UW (30.9 ± 4.48 vs. 35.3 ± 5.49 years, p=0.022), NW (34.2 ± 4.25 vs. 36.3 ± 4.84 years, p<0.001), and OW (33.8 ± 4.89 vs. 36.3 ± 5.31 years, p=0.009) groups. Although a similar trend could be observed, however, the age difference did not reach statistical significance in the OB group between the successfully conceived and not conceived subgroups (33.7 ± 4.87 vs. 36.3 ± 5.81 years, p=0.055, respectively) (**Figure 2A**).

Considering FSH, LH, and AMH hormone levels in each BMI category, a statistically significant difference was observed only in the NW category between the groups that conceived and those that did not: FSH was significantly lower (7.8 ± 2.99 vs. 8.6 ± 3.50 IU/L, p=0.032) and AMH (2.87 ± 2.40 vs. 2.28 ± 2.01 pmol/L, p=0.021) was higher in the successfully conceived women (**Figures 2B–D**).

Across any BMI category, according to the duration of infertility, the number of unsuccessful IUI, duration of stimulation, number of discontinued cycles, total number of transferred embryos, endometrial thickness at ovulation/on the day of puncture/at the time of embryo transfer, the parameters of semen analysis there were no statistically significant differences observed between the groups that achieved pregnancy and those that did not.

The number of follicles was found to be significantly higher in NW (7.3 ± 2.90 vs. 6.5 ± 2.95, 0.015) and OW (7.6 ± 3.66 vs. 6.2 ± 2.46, p=0.029) women who conceived compared to those who did not conceive (**Figure 2E**).

The age of male partners differed significantly only in the NW category between the two subgroups: the average age of partners of women who conceived was significantly younger than those of women who did not conceive (37.6 ± 5.94 vs. 39.4 ± 6.17 years, p=0.010) (**Figure 2F**).

The embryo score of the best transferred embryo was significantly lower in the case of non-pregnant subgroup within both the NW (p<0.001) and OW (p<0.001) categories (**Figure 2G**).

### Increasing BMI, decreasing serum FSH and LH; a larger population analysis

**Figure 3:**
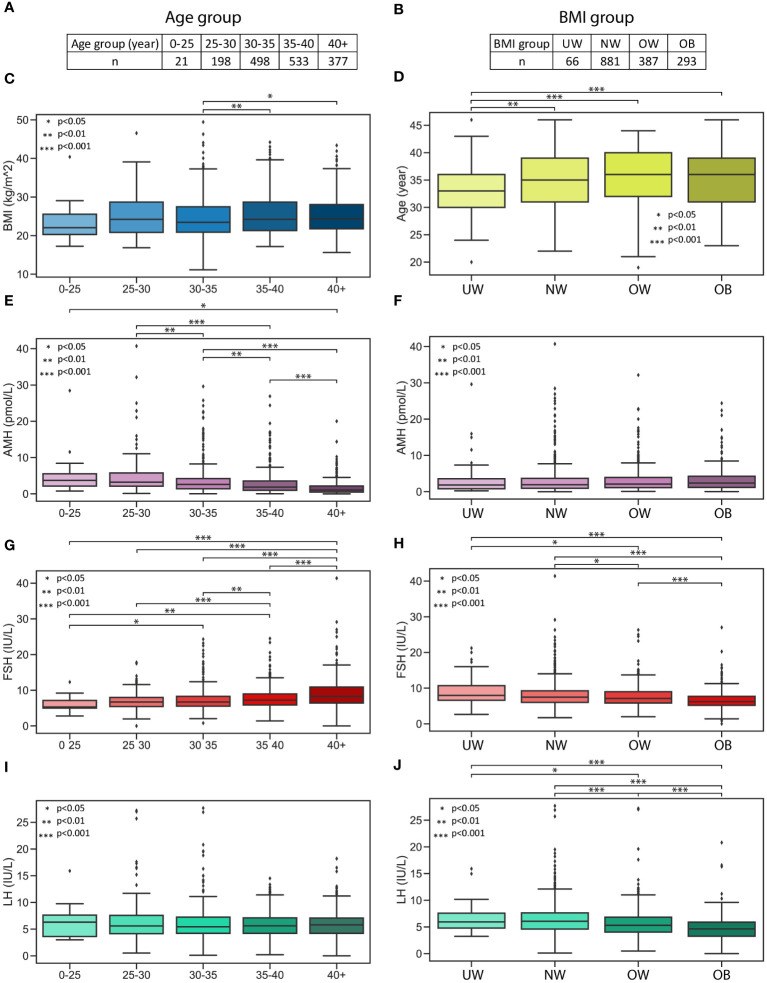
AMH, FSH and LH hormone levels in age and BMI groups. AMH, FSH and LH hormone levels in age and BMI groups. **(A)** Age categories and the number of measurements. **(B)** BMI categories and the number of measurements. **(C)** Boxplot represents the distribution of BMI in age categories. **(D)** Boxplot represents the distribution of age in BMI categories. **(E)** Boxplot represents the AMH levels in age groups. **(F)** Boxplot represents the AMH levels in BMI groups. **(G)** Boxplot represents the FSH levels in age groups. **(H)** Boxplot represents the FSH levels in BMI groups. **(I)** Boxplot represents the LH levels in age groups. **(J)** Boxplot represents the LH levels in BMI groups.

The increase in BMI was accompanied by a decrease in the levels of follicle-stimulating hormone (FSH) and luteinizing hormone (LH) in the serum. To verify this surprising and interesting correlation, we enlisted a larger number of cases. Extracting cycle initiation data from the Institute of Reproductive Medicine, Albert Szent-Györgyi Medical Centre, University of Szeged, between January 1, 2020, and November 30, 2023, with a focus on ensuring that each patient is represented only once with their initial data, we examined 1627 cases to explore the relationship between BMI, FSH, and LH. This expanded dataset includes age (year), height (cm), weight (kg), FSH (IU/L), LH (IU/L), and AMH (pmol/L), allowing us to investigate changes in hormone levels based on age and BMI categories. Five age groups were established as follows: 0-25, 25-30, 30-35, 35-40, 40+, and BMI categories were determined as described earlier. Important to note that in both cases, a single category had fewer records; the “younger than 25 years old” category held 21 cases, while the underweight category consisted of 66 cases (**Figures 3A, B**). Initially, an examination was conducted to explore the potential connection between age and BMI groups. A minor but significant increase in BMI was found between the 30-35 and 35-40 (p>0.01) as well as the 30-35 and 40+ (p>0.05) categories, with no significant differences observed in any other combinations (**Figure 3C**). In the case of BMI groups, the underweight category was significantly younger than any other category (NW p>0.01, OW p>0.001, OB p>0.001) (**Figure 3D**). Considering these observations, we concluded that with ageing, the BMI slightly increases; however, this does not have a striking effect on BMI categories in our dataset. Our analyses revealed the anticipated changes, with the serum levels of AMH significantly and progressively decreasing in older age groups (**Figure 3E**). Interestingly, AMH levels showed no significant changes between the BMI groups (**Figure 3F**). In contrast to AMH levels, the FSH levels consistently and significantly increase with the progression of time (**Figure 3G**). Meanwhile, with higher BMI, FSH levels are significantly lower (**Figure 3H**). LH levels showed no significant changes in age groups (**Figure 3I**). Meanwhile, LH levels are significantly lower in higher BMI groups (**Figure 3J**).

### Correlations in the whole population

**Figure 4:**
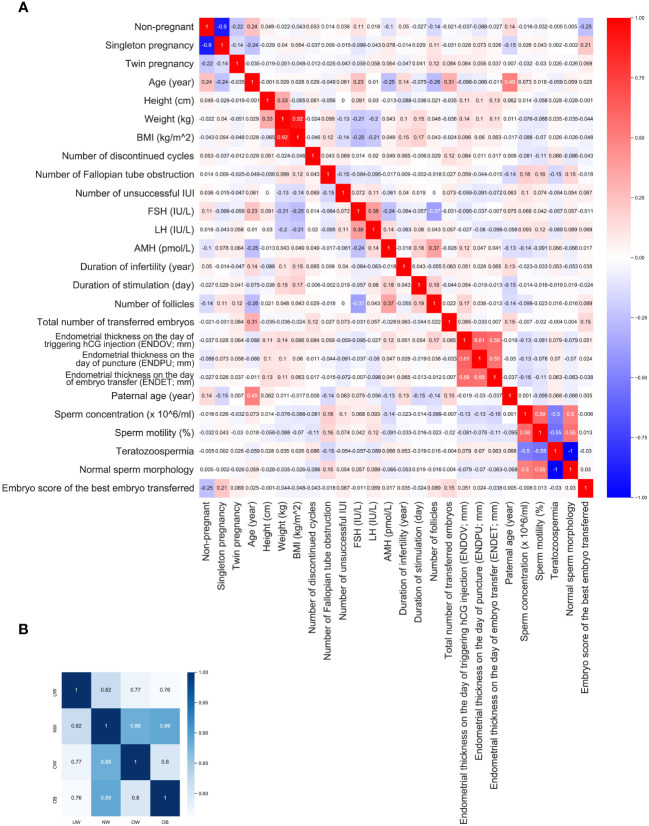
Correlations. **(A)** heatmap represents the correlations within the entire dataset, with rho values rounded to three decimals. **(B)** heatmap illustrates the correlation between BMI groups correlations.

Since the analyzed medical data has a relatively high number of dimensions (n=26), the correlation between these categories was calculated in order to find potentially interesting connections (**Figure 4A**, **Supplementary Table 2**).

BMI and weight correlated negatively with FSH (r=-0.252, p<0.001, r=-0.206, p<0.001, respectively) and LH (r= -0.213, p<0.001; r= -0.195, p<0.001, respectively). A weaker negative correlation could be found between BMI and unsuccessful IUI (r=-0.13, p= 0.00), and also between weight and unsuccessful IUI (r=-0.13, p= 0.003). A weak positive correlation was found between BMI and the duration of infertility (r= 0.146, p<0.001), and the duration of stimulation (r= 0.173, p<0.001). Not surprisingly, age correlated positively with the non-pregnancy (r=0.236, p<0.001;***) and negatively with pregnancy (r= - 0.236, p<0.001;***) rate. Furthermore, maternal age correlated positively with serum FSH level (r=0.229, p<0.001;***), with the duration of infertility (r= 0.143, p<0.001;***), and negatively with AMH level (r= - 0.249, p<0.001;***), and with the number of follicles (r= -0.263; p<0.001;***). FSH correlated negatively with the number of follicles (r: -0.369, p<0.001;***). AMH level correlated positively with the duration of stimulation (r=0.178, p<0.001;***), and with the number of follicles (r=0.367, p<0.001;***).

For further investigation correlation in each BMI group was calculated. Additionally, the correlation between the BMI groups was also calculated, showing high similarity between categories. NW group is more similar to UW (r=0.817, p<0.001; ***), OW (r=0.880, p<0.001; ***) and OB (r=0.889, p<0.001; ***) group than any other group. Meanwhile UW is the most diverse group (**Figure 4B**).

### Correlations in the UW group

BMI showed no correlation with any variable. Age correlated negatively with unsuccessful IUI (r= -0.374, p= 0.035), and with the number of follicles (r= -0.432, p= 0.0135). The number of discontinued cycles correlated positively with AMH (r= 0.434, p= 0.013), and negatively with ENDET (r= -0.354, p= 0.047). Unsuccessful IUI correlated negatively with the paternal age (r= -0.375, p= 0.035). The number of follicles correlated negatively with paternal age (r= -0.423, p= 0.016). ENDOV correlated negatively with the maternal age (r= -0.353, p= 0.048). FSH correlated positively with LH (r=0.553, p=001), and negatively with AMH (r=-0.350, p=0.050), and the number of follicles (r=-0.457, p=0.009). Unsuccessful IUI has correlated negatively with the embryo score of the transferred best embryo (r=-0.370; p=0.037).

### Correlations in the NW group

Age correlated positively with the non-pregnancy (r=0.221, p<0.001;***), FSH (r= 0.267, p<0.001;***), duration of infertility (r=0.122, P=0.028), the total number of transferred embryos (r=0.346, p<0.001;***), LH (r=0.130, p=0.019), and with the number of unsuccessful IUI (r=0.117, p=0.036) and negatively with singleton pregnancy (r= - 0.219, p<0.001;***) rate, AMH (r=- 0.243, p<0.001;***), and number of follicles (r=–0.286, p<0.001;***). The embryo score of the transferred best embryo correlated positively with the number of follicles (r=0.113, p=0.043), negatively with teratozoospermia (r=-0.133, p=0.016), also positively with the singleton pregnancy (r=0.227, p<0.001;***), and as expected, negatively with the non-pregnant status (r=-0.264; p<0.001;***). FSH correlated positively with LH (r=0.397, p<0.001;***), negatively with the number of follicles (r=-0.408, p<0.001;***). LH correlated negatively with BMI (r=-0.188, p<0.001;***), positively with AMH (r=0.153, p=0.006). AMH correlated positively with the number of follicles (r=0.371, p=0.006), and duration of stimulation (r=0.114, p=0.040). The number of follicles correlated positively with the duration of stimulation (r=0.211, p<0.0001), with the singleton pregnant state (r=0.111, p=0.046), and negatively with the non-pregnant state (r=-0135, p=0.015).

### Correlations in the OW group

Weight correlated positively with ENDOV (r=0.242, p=0.009), and with ENDET (r= 0.221, p= 0.016). Duration of stimulation correlated positively with AMH (r= 0.384, p<0.001; ***), and negatively with the age (r= -0.253, p= 0.006).

### Correlations in the OB group

FSH correlated negatively with ENDPU (r= - 0.311, p=0.010 and with the number of follicles (r=-0.311, p= 0.011). AMH correlated positively with ENDOV (r=0.2281, p=0.022). The embryo score of the transferred best embryo correlated only with the number of Fallopian tube obstruction (r=0.263, p=0.033). ENDOV correlated positively with the duration of stimulation (r=0.282, p=0.022). ENDOV also correlated positively with the number of follicles (r=0.257, p=0.037).

### BMI and the development of endometrium

**Figure 5:**
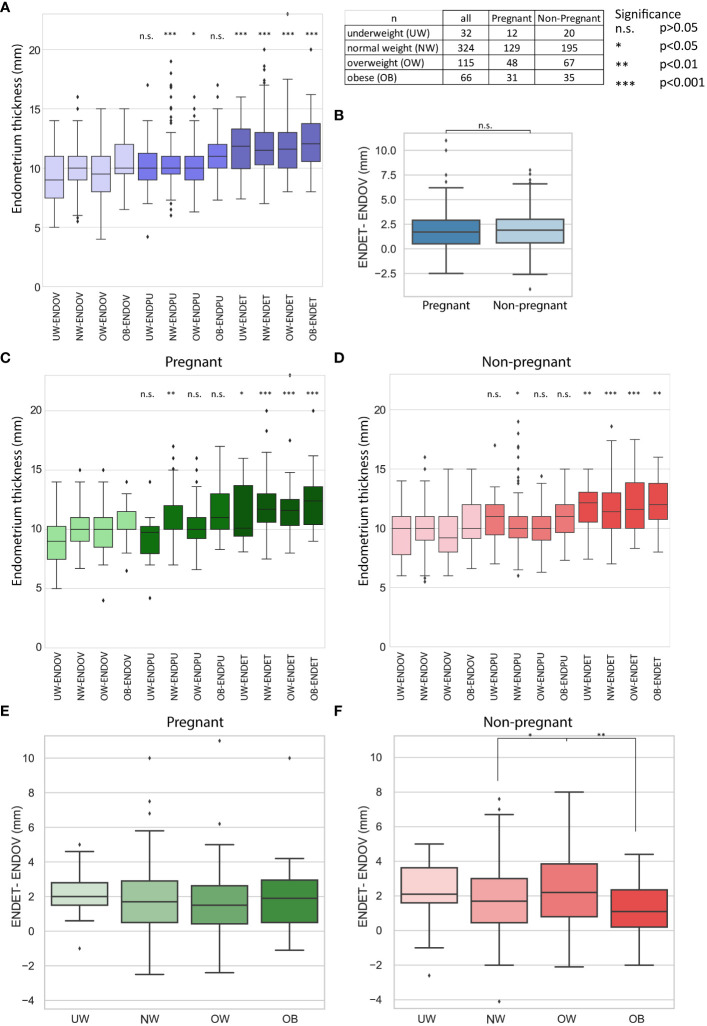
Results of endometrial thickness measurement. Sample numbers and significance boundaries are available in the top right corner. **(A)** The boxplot represents alterations in endometrial thickness at three measurement times (ENDOV, ENDPU, ENDET). Significance is highlighted in the relation of ENDOV and the corresponding BMI categories in ENDPU and ENDET. **(B)** The boxplot represents the difference in endometrial thickness between the ENDOV and ENDET measurements in non-pregnant and pregnant categories. **(C, D)** Boxplots illustrate alterations in endometrial thickness across three measurement times (ENDOV, ENDPU, ENDET) for both non-pregnant and pregnant groups. Significance is emphasized in the relationship between ENDOV and the corresponding BMI categories in ENDPU and ENDET. **(E, F)** Boxplots illustrate the difference in endometrial thickness between ENDOV and ENDET measurements across BMI categories for non-pregnant and pregnant groups. Only significant differences are indicated. Additional statistical information is available in the *.xlsx file.

The health and thickness of the endometrium have a significant impact on the success of embryo implantation. Endometrial thickness can be measured using ultrasound, and these measurements can be utilized to assess the effectiveness of hCG treatment. Endometrial thickness was measured at three time points - specifically, at the time of triggering hCG injection, on the day of puncture, and at embryo transfer - by a single medical expert. The data for endometrial thickness on the day of triggering hCG injection (ENDOV), on the day of puncture (ENDPU), and at embryo transfer (ENDET) are presented in **Figure 5** based on BMI categories and pregnant/non-pregnant subgroups.

Investigating the BMI groups in each category a significant thickening was observed (**Figure 5A**). Examining the development of the endometrium in response to treatment involved subtracting ENDOV values from ENDET values (**Figure 5B**). These observations suggest that endometrial thickening is generally present in the studied population.

For further investigation, the non-pregnant and pregnant categories were utilized. Based on the BMI groups, it was observed that the significance is less prominent compared to the whole dataset in the case of underweight ENDOV and ENDET and obese ENDOV and ENDET categories in the case of non-pregnant group, and underweight ENDOV and ENDET in the case of pregnant group (**Figures 5C, D**). In the obese group, p<0.001 significance remained between ENDOV and ENDET in pregnant category. These alterations primarily affected the least represented groups, indicating that an increase in case numbers within these groups would be beneficial for future studies.

Next, the investigation focused on the changes of the endometrium thickness by subtracting ENDOV values from ENDET values. Within the pregnant group, no significant difference was observed among BMI categories regarding the development of endometrial thickness. In the non-pregnant group, significant differences were found between the NW – OW (p<0.001) and OW - OB (p<0.001) categories (**Figures 5E, F**). This suggests that the growth of the endometrium does not vary greatly among different BMI groups.

When categorized by BMI, no significant difference was observed in the comparison between pregnant and non-pregnant groups. The average percentage growth in endometrial thickness for each BMI category is demonstrated in **Table 3**. However, within the OB category of the non-pregnant group, the endometrium is noticeably thinner than in the pregnant group, although the difference is not significant. The lack of significance may be attributed to the lower sample size, with 35 non-pregnant patients and 30 pregnant subjects; an interesting trend might emerge with an increase in sample size. Keeping in mind the difference between the significance of ENDOV and ENDET overall thickness in OB groups, as in pregnant it is p<0.001 and in non-pregnant p<0.01.

**Table 3:** Average percentage growth in endometrial thickness for each BMI category.

BMI category	Number of Pregnant patients	Number of Non-pregnant patients	Growth of the endometrium (%) Pregnant Group	Growth of the endometrium (%) Non-pregnant Group
**UW**	12	20	26.90	27.30
**NW**	129	195	18.95	18.62
**OW**	48	67	20.60	27.72
**OB**	31	35	19.08	14.56

This data raises the possibility that in OB group the endometrium thickening is less efficient in the non-pregnant group, which might affect the success of pregnancy, yet further investigations are needed to confirm this hypothesis. Another noteworthy finding is the absence of significant differences between the pregnant and non-pregnant groups, raising the possibility of the role of endometrial quality next to quantity.

**Figure 6:**
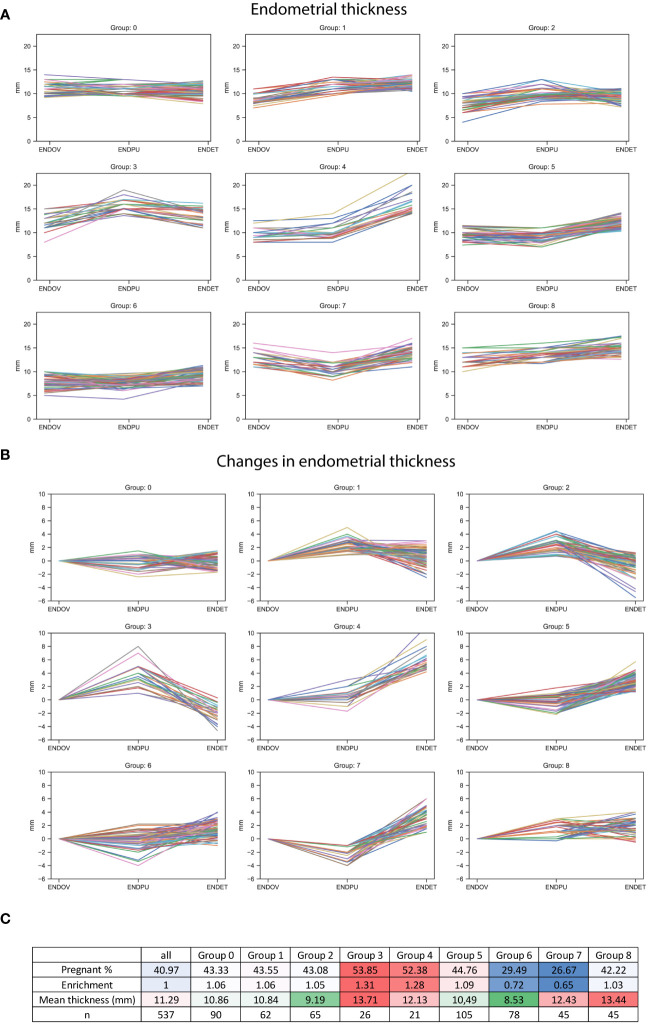
Endometrial thickness in k-means clusters. **(A)** Line graphs represent endometrial thickness in nine categories, using the 3 measurement times. **(B)** Line graphs represent the changes in endometrial thickness between the measurements, in the nine groups. **(C)** Table describing the nine categories, including the percentage and the enrichment of pregnant outcomes (higher values represented with a red, lower values a blue background), the mean overall thickness measured in each category (higher values represented with a red, lower values with a green background), and the number of the members of the group.

Next, k-means clustering (k=9) was utilized to determine patterns in the thickening of endometrium. For determining of the clusters the measured endometrial thickness, and the changes between the measurement points were used (**Figures 6A, B**). Based on the clustering, two groups (Group 3 and 4) exhibited a significantly higher rate of pregnancy, while two other groups (Group 6 and 7) demonstrated a considerably lower rate of pregnancy (**Figure 6C**). Group 4 showed a consistent trend of endometrial thickening across all measured time points. Interestingly, a peak in endometrial thickness was observed in Group 3 at the ENDPU time point. Group 6 showed only a limited thickening, in contrast to Group 7 which has a minimum of endometrial thickness at ENDPU measurements. Group 3 and Group 7 exhibited an interesting pattern, both indicating the potential importance of endometrial thickness at the ENDPU time point. In Group 3, endometrial thickness decreased up to ENDET; however, the pregnancy rate was higher in this group. Meanwhile, Group 7 exhibited the opposite trend, with endometrial thickening at ENDET, yet pregnancies were less prominent in this group. Regarding the mean thickness of endometrium between the groups, Group 2 and Group 6 had the thinnest endometrium. Interestingly, in Group 2, this was not associated with a decrease in pregnancies. In Group 8, the situation was reversed; the mean endometrial thickness was high, yet the percentage of pregnancies did not increase considerably. Moreover, Group 7 showed a relatively high mean value, meanwhile having the lowest pregnancy rate. Taken together investigating these clusters showed that thin endometrium is not preferable for pregnancy, yet not determining exclusively the success of pregnancy.

### Endometrial thickness at the measured three time points for determining pregnancy

**Figure 7:**
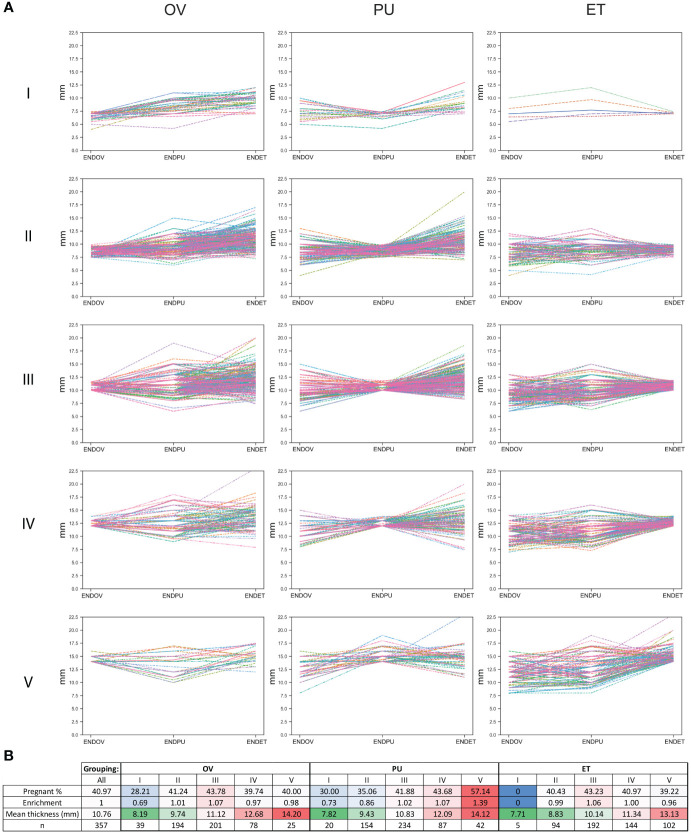
Endometrial thickness categorized based on thickness at different measurement time points. **(A)** line graphs depict endometrial thickness in five categories (I, II, III, IV, V), across three measurement times (OV, PU, ET). **(B)** table describing the categories, including the percentage and enrichment of pregnant outcomes (higher values represented with a red, lower value with a blue background), the mean overall thickness measured in each category (higher values represented with a red, lower value with a green background), and the number of members in each group.

As a follow-up, the data were categorized based on the endometrial thickness at the three different time points (**Figure 7A**). Five groups were generated for each measurement time: Group I < 7.5 mm, 7.5 mm ≤ Group II < 10 mm, 10 mm ≤ Group III < 12 mm, 12 mm ≤ Group IV < 14 mm, Group V ≥14 mm. At all measurement times, Group I, representing the thinnest category, exhibited a decreased rate of pregnancy outcomes (**Figure 7B**). Interestingly, there were no considerable differences in the rate of pregnancies in II, IV and V Groups at ENDOV and ENDET measurements. There was a slight increase in the rate of pregnancies in Group III, observed at both ENDOV and ENDET time points (**Figure 7B**). At the ENDPU measurements, a thicker endometrium was observed to be more favorable for pregnancy (**Figure 7B**), as the ratio of pregnant outcomes increased with greater endometrial thickness.

As summary, we found that thin endometrium is not preferable for pregnancy, yet endometrial thickness is not equally important at different time points. Intermediate endometrial thickness is slightly better at OV and ET, but thicker endometrium is superior at PU. This data suggests that at ET endometrial quality is more important than quantity.

### Support vector machine (SVM) model

**Figure 8:**
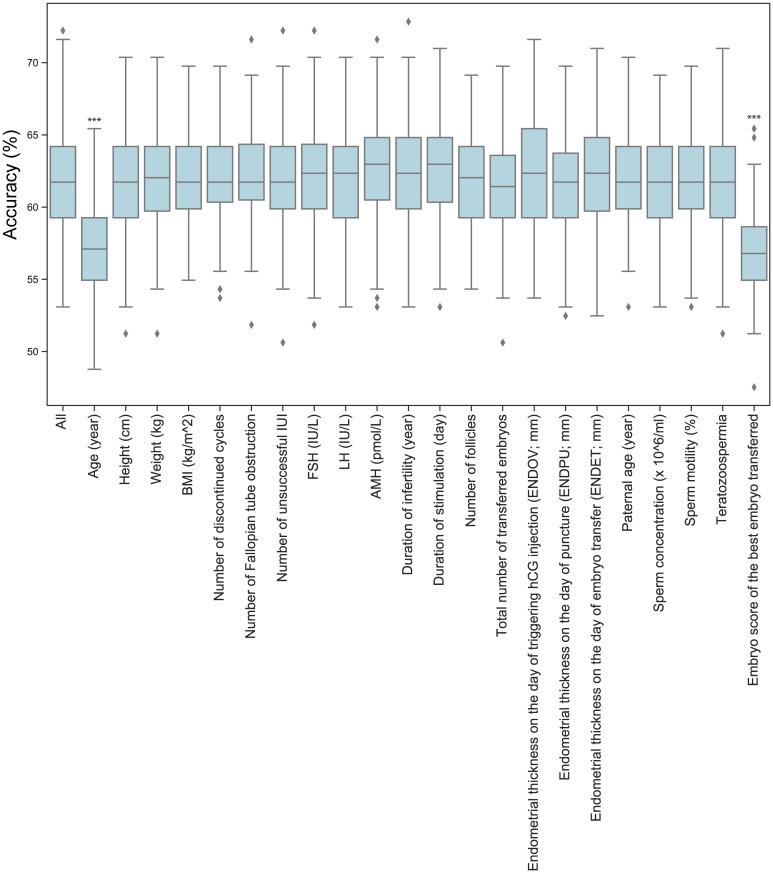
The effect of omitting dimensions on SVM models accuracy. The boxplot illustrates the accuracy of optimized Support Vector Machine (SVM) models using 100 different random states. The unused data for model training is listed on the x-axis. Significant differences in performance compared to the full dataset (All) are marked by *** p<0.001.

**Figure 9:**
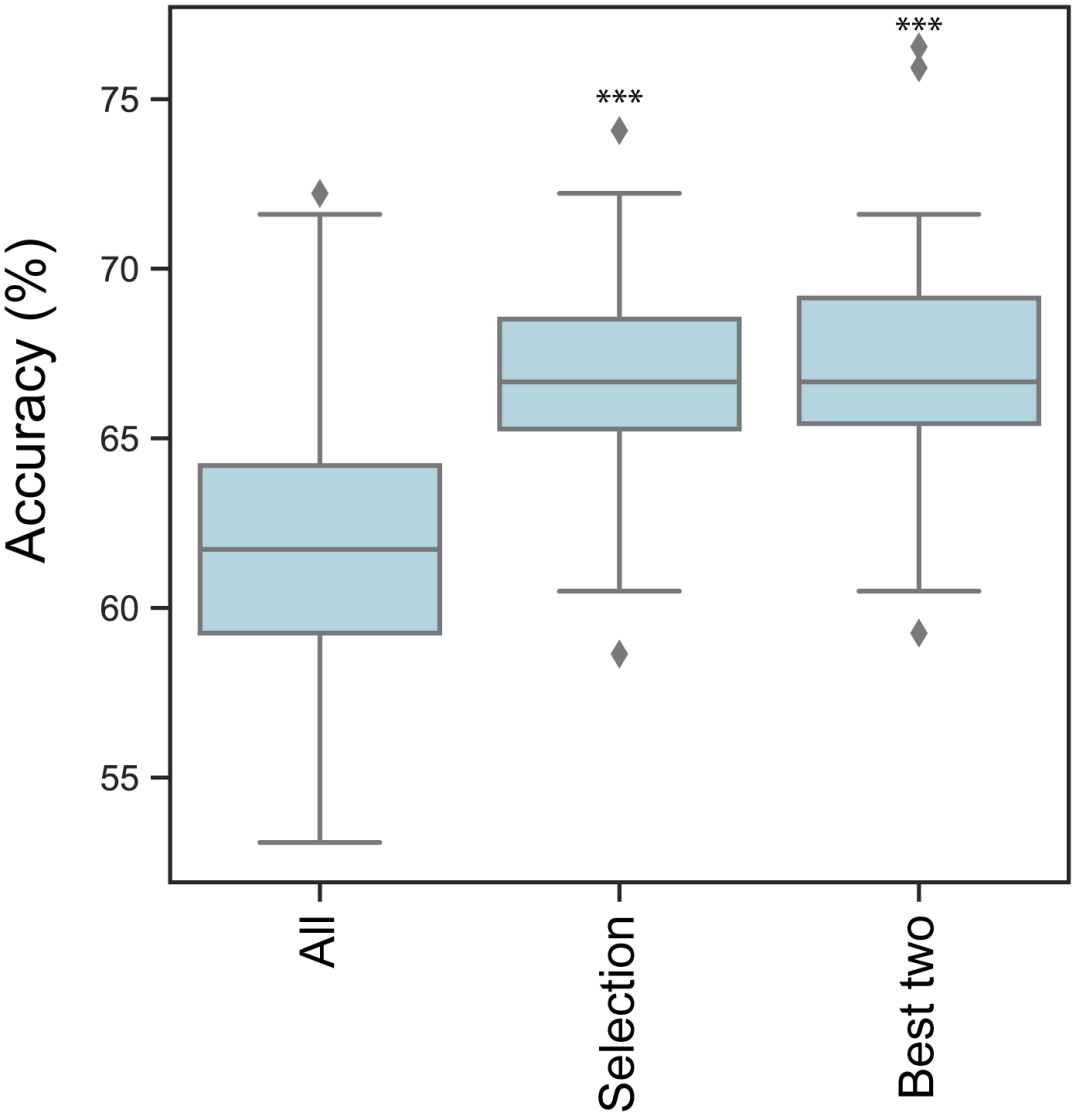
SVM models accuracy. Boxplot represents the mean accuracy of optimized Support Vector Machine (SVM) models through 100 different random state iterations. ‘All’ group SVM calculations contain all 22 dimensions, ‘Selection’ group contains age, total number of transferred embryos, endometrial thickness on the day of puncture and embryo quality, The Best two group contains age and embryo quality scores. Significant differences in performance compared to the full dataset (All) are marked by *** p<0.001.

We aimed to utilize advanced statistical methods to predict potential pregnancies based on medical data gathered from 537 patients, comprising 317 cases of unsuccessful treatment and 220 cases of successful treatment. A similar approach was tested earlier by Liu et al. (21). We chose to build Support Vector Machine (SVM) models, SVM works relatively well on smaller datasets and these models can be easily optimized. Liu et al. utilized six dimensions for predicting the success of embryo transfer, including age, body mass index, endometrial thickness on the day of progesterone treatment, the rate of good quality embryos, estradiol and progesterone levels of the serum on the day of embryo transfer, reaching 55% accuracy in the SVM model predictions. The conclusion of their work suggested to seek for more predictors to achieve better models. In this study we used 22 dimensions (**Figure 8**), we used 30% of the total data for training the model and 70% for testing. To ensure equal weighting for each dimension, the data was standardized prior to the training of the model. Iterating through 100 different random states, statistics were generated for the accuracy of the models. For each iteration, the best parameters for C (0.1, 1, 10, 100, 1000) and gamma (1, 0.1, 0.01, 0.001, 0.0001) values were sought. The focus was on the accuracy of each iteration. The average accuracy of predictions was 61.71%. The conclusion drawn is that our model outperforms previous models; however, there is insufficient data to confidently predict the outcome of embryo transfer. There is still room for improvement and screening for further predictors could have a positive impact in ameliorating the models. To investigate further which dimensions have a higher impact, we omit them one by one and checked the performance change in the average accuracy of the models (**Figure 8**). Minimal, not significant performance loss was observable in the absence of the total number of transferred embryos (average accuracy: 61.48%) and endometrial thickness on the day of puncture (ENDPU) (average accuracy: 61.61%). Meanwhile, the highest performance loss occurred when omitting age (average accuracy: 57.5%) and embryo quality (average accuracy: 56.64%); the absence of these data significantly decreased (p<0.001) the accuracy of the SVM model. The other data categories did not exhibit significant changes in predictions accuracy, however, omitting them slightly increased the performance of the model. We determined the variance inflation factor (VIF) for each dimensions to assess the potential influence of multicollinearity. Only 3 instances showed a value larger than five, namely the Height, Weight, and BMI. Based on this result, we investigated the simultaneous omission of these 3 dimensions from the model, and found a slight increase in the model accuracy (**Supplementary Table 3**). Based on these observation, further tests were conducted incorporating only maternal age, total number of transferred embryos, endometrial thickness on the day of puncture (ENDPU), and embryo quality into the analyses (**Figure 9**). The SVM model’s average accuracy significantly increased to 66.79% (p<0.001). Next, only the two best estimators, age, and embryo quality were utilized, yielding an average of 66.98% (p<0.001) accuracy (**Figure 9**).

To further examine the observed differences, we investigated the Receiver Operating Characteristic (ROC). The ROC curve of linear SVM models was tested using 10 times stratified K-fold cross-validation. The same three sets of data as before were employed (**Supplementary Figure 1**). The selected categories were found to enhance the performance of these models, resulting in outcomes similar to those observed previously.

## Discussion

### BMI categories and clinical data the whole population

Obesity is a major contributing factor to various underlying causes associated with infertility (22). Regarding the literature women with obesity experience poorer reproductive outcomes, irrespective of the mode of conception, which includes natural conception, pregnancies resulting from ovulation induction, IVF, ICSI, and ovum donation programs. Furthermore, a direct correlation has been established between a higher BMI and an unfavourable fertility prognosis (23–27). A recent meta-analysis of 53 studies comprising 1,445,406 treatment cycles indicated that an elevated BMI was associated with a mild negative impact on outcomes for women undergoing IVF or ICSI (28). Obesity is linked to anovulation independently of PCOS. Women with a BMI higher than 27 kg/m^2^ face an elevated risk of anovulatory infertility compared to those with a BMI within the normal range (relative risk, 3.1 [95% CI, 2.2-4.4]) (29).

Regarding the onset of menstruation, a minimum amount of fat mass is necessary to maintain ovulatory function. Romanski et al. (30) in a retrospective cohort study of infertile patients found that infertile women who are mildly or significantly underweight exhibit comparable pregnancy and live birth rates to normal-weight patients following IVF treatment. Moreover, underweight patients do not face an elevated risk of preterm delivery (<37 weeks), Caesarean delivery, or having a neonate with a low birth weight (30).

It is important to note that the BMI distribution of female patients undergoing IVF deviates from the BMI distribution in the general Hungarian population; the overweight/obese female population is notably underrepresented in our data (OW: 21.41% vs. 29.12% and OB: 12.29% vs. 25.64%). This discrepancy was surprising to us as well, and its cause should definitely be considered multifactorial. The clear reasons are questionable, with primary consideration given to the combination of psychological, relationship dynamical, societal, and epidemiological factors. For further verification and identification of these aspects, a public health study is planned.

### AMH independence from BMI alterations; increasing BMI, decreasing serum FSH and LH

From birth to adolescence, AMH levels gradually increase, plateauing, and suggesting distinct follicle dynamics in children compared to adults, with the decline in serum AMH becoming apparent only after the age of 25 (31, 32). Our analyses also revealed the anticipated changes, with the serum levels of AMH significantly and progressively decreasing in older age groups (**Figure 3E**). Regarding AMH levels, our study conducted on the smaller primary sample size (n=537) revealed a weaker significant difference (p<0.05; *) between NW and OB (**Figure 1F**). However, this difference was not present in the analysis of the larger dataset (n=1627) (**Figure 3F**). This aligns with a recent narrative review (33) of 13 studies on AMH in women, which could not demonstrate any clear impact of obesity on AMH levels. More studies have not shown an association between obesity and AMH in premenopausal female subjects (34) or healthy female patients (35). In contrast to our findings, a meta-analysis of 4,055 records and 45 original studies, evaluating the independent associations of BMI or obesity with ovarian reserve markers in reproductive-aged women, has revealed a significant reduction in AMH levels in female subjects with obesity compared to women without obesity (36).

In contrast to AMH levels, the FSH levels consistently and significantly increase with the progression of time, as demonstrated in our investigation (**Figure 3G**). The levels of circulating FSH experience significant alterations primarily attributed to the diminishing negative feedback from inhibin, as well as androgens and estrogens in ageing females (37). For instance, increased circulating FSH levels serve as diagnostic indicators of menopause in women. Meanwhile, with higher BMI, FSH levels are significantly lower (**Figure 3H**). Furthermore, the aforementioned meta-analysis (36), similar to our findings, revealed a negative correlation between BMI and FSH, which could only be confirmed in the fertile non-PCOS subgroup; however, PCOS and fertility status did not appear to affect these associations.

LH levels showed no significant changes in age groups (**Figure 3I**). Meanwhile, LH levels are significantly lower in higher BMI groups. This association has already been described in normally menstruating women (38, 39). In a recent review (40) the various mechanisms contributing to female obesity-related secondary hypogonadism have been discussed. Obesity is linked to an increase in leptin levels (41), potentially resulting in hypothalamic leptin resistance (42) and a reduction in GnRH pulsatile amplitude (39) and LH levels (39). Lower LH levels may occur due to increased clearance of endogenous LH in women with obesity (43, 44), as well as a diminished pituitary response to GnRH (45). Androgen levels are increased in women with obesity (46), and markedly elevated levels could contribute to a reduction in LH levels (47). Obesity is associated with an increase in inflammatory markers (45), which can also contribute to the observed reduction in LH levels in women with obesity.

Our analyses showed that both age and BMI have an effect on the baseline hormone levels. AMH levels change with age but are not affected by BMI. Meanwhile, LH levels remain more constant across age groups, yet they are lower in higher BMI groups. Age and BMI can have a seemingly antagonistic effect, as observed in the case of FSH, where FSH increases in age groups and decreases in BMI groups. These connections support the evolutionary selective role of nutritional status. As far as we know, there has not been a description of the inverse correlation between BMI and LH, FSH in the infertile female population so far. Furthermore, our results are the first in the context of the Hungarian infertile female population.

### Correlations in the whole population and in BMI categories

Overall, a common correlation trend could not be identified within the examined four BMI categories. The investigation of the entire studied population, without subgroup analysis, identified several correlations. BMI demonstrated a negative correlation with the previously discussed FSH and LH levels, and a weaker negative correlation could be found between BMI and unsuccessful IUI. The cause of the latter is certainly considered multifactorial. Additionally, BMI correlated positively with the duration of infertility and stimulation. The latter is also not surprising, as several studies on women undergoing IVF found that the duration of stimulation was significantly higher in the obese group (48, 49) when compared to the normal BMI group.

In the whole population, maternal age correlated positively with the duration of infertility. This connection also encourages us to increase fertility awareness on a national level is important to ensure that patients reach infertility specialists in a timely manner, preferably at a younger age. In the whole population, maternal age, not surprisingly, also correlated positively with FSH level and negatively with AMH and the number of follicles.

Considering the found correlations among the four BMI categories, significant overlaps and similar trends were not identifiable. Highlighting the most interesting relationships from individual categories, in the UW category, while BMI did not show any correlation, ENDOV negatively correlated with maternal age, and ENDET correlated with the number of discontinued cycles. Furthermore, the baseline FSH level showed a positive correlation with LH, AMH, and negative correlation with the number of follicles. These latter three observations proved to be true even for the largest sample-sized NW category. The negative correlation between FSH and AMH is not unexpected in infertile women undergoing IVF treatment, as it had already been documented in the literature (50), although with a significantly smaller sample size (n=81) compared to our study (n=537). Furthermore, LH showed a negative correlation with BMI, which can be attributed to the reasons discussed earlier.

In OW category, the weight correlated positively with ENDOV, and with ENDET. Additionally, the duration of stimulation correlated positively with AMH, and negatively with the maternal age. This aligns with the fact that typically, when maternal age is higher, a higher dose of recombinant FSH is employed, leading to a more rapid response.

Only in the OB category, the embryo score of the transferred best embryo correlate with the number of Fallopian tube obstruction. Patients, theoretically only having a tubal blockage, are considered to have the best prognosis. Additionally, in OB category FSH correlated negatively with ENDPU and with the number of follicles. AMH correlated positively with ENDOV. This latter connection is also not surprising, as rapid follicular development, early puncture, and short stimulation lead to poorer outcomes (51, 52). ENDOV correlated positively with the duration of stimulation. ENDOV also correlated positively with the number of follicles.

### BMI and the development of endometrium

#### Endometrial thickness at the measured three time points for determining pregnancy

The study of the endometrium’s health and thickness is intriguing due to its potential impact on the success of embryo implantation. Protocol-wise, ultrasound measurement of endometrial thickness is always conducted on the day of hCG injection and embryo transfer; however, it is not routinely performed on the day of the follicular puncture. In this study, a single medical specialist assessed the endometrial thickness at three different instances. These instances were specifically the time of hCG injection, the day of follicular puncture, and immediately preceding the embryo transfer. Through this data collection approach, we were able to conduct a comprehensive investigation into the thickening of the endometrium, its dynamics, and the success of embryo transfer. The findings from our data demonstrated a significant thickening observed between ENDOV and ENDET. This trend was observable in both the pregnant and non-pregnant groups across all four BMI groups. Regarding the absolute growth of endometrium among different BMI groups, there were no significant differences in the pregnant group. However, in the non-pregnant-group, thickening in the OB category was the least, which was significantly smaller than in the OW group. This data suggests that the thickening of the endometrium in the non-pregnant group may be less efficient within the OB category.

Interestingly, we found no significant differences between the pregnant and non-pregnant group in regard of endometrial thickness. This finding is unexpected, considering that the proper state of the endometrium is crucial for successful embryo implantation. To address this issue, k-means clusters (k=9) were created, revealing various patterns of endometrial growth. Regarding mean endometrial thickness, there were groups with thinner or thicker endometrium. However, it did not considerably influence the pregnancy in every group. Despite overall low endometrial thickness Group 2 (9.19 mm) had a 43.55% pregnancy rate, while Group 6 (8.53mm) only had a 29.49% rate. Similarly, Group 8 had a thick endometrium (13.44 mm) with a 42.22% pregnancy rate, and Group 3 also had a thick endometrium (13.71 mm) but with an increased pregnancy rate of 53.85%. Moreover, Group 4 (12.13 mm) and Group 7 (12.43 mm) had a similar mean endometrial thickness, yet exhibited very different pregnancy rates: 52.38% and 26.57%, respectively. These findings might explain why we could not identify significant differences in the whole population. However, it is worth noting that our findings revealed a low pregnancy rate in cases where the endometrial thickness was high. Nonetheless, we did not observe any group with a thin endometrium that displayed an outstandingly enhanced fertility rate. Moreover, Group 6 exhibits a pregnancy rate that is more than 10% lower than the overall population. Interestingly, this group also demonstrates the lowest average endometrial thickness, measuring at 8.53 mm. This finding strongly indicates that endometrial thickness below this value may pose a risk for successful embryo implantation.

There were two groups that had a considerably increased rate of pregnancy (Group 3 – 53.85%, Group 4 – 52.38%). Meanwhile two groups had a considerably decreased rate of pregnancy (Group 6 – 29.49%, Group 7 – 26.67%) compared to the total population (40.97%). In Group 3, a pattern could be observed where ENDPU has the highest value. This pattern was somewhat similar in Group 2 where endometrium was thinner; yet, pregnancy rate (43.08%) slightly exceeded the population average (40.97%). Curiously, Group 7 exhibited a contrast as it showed the minimum value of ENDPU, which was opposite to Group 3. Group 6, with the lowest mean endometrial thickness (8.53 mm) showed a more diverse pattern with limited changes in thickness. There was also a peculiar similarity between Group 4 (52.38%) and 7 (26.67%) regarding patterns, as in both groups, there was a steep elevation between ENDPU and ENDET. The difference between these groups was a considerable decrease between ENDOV and ENDPU in Group 7, which was less characteristic to Group 4. Taken together the data suggests the dynamics of endometrium contribute to the success of embryo implantation. The decline in endometrial thickness from the day of triggering hCG injection (OV) to the time point of the follicular puncture (PU) phase is associated with an increased risk, while an elevation in endometrial thickness is advantageous for the successful development of pregnancy. These results also prompted a more thorough analysis of endometrial thickness. Five categories based on the thickness of three time points were created. In all three measurement times - OV, PU and at the time of embryo transfer (ET) - within the thinnest category (I, <7.5 mm) the pregnancy rate was low (<30%). This means at any time point, a low endometrial thickness poses a risk at the success of embryo implantation. Surprisingly, though in the case of OV and ET time points, the other categories showed minimal differences between the pregnancy rates. Moreover, at these time points category III had the highest pregnancy rate (>43%), which was marginally above the population average (40.97%). In contrast to this, at PU measurement, the rate of pregnancy gradually increased with the thickness of the endometrium, reaching 57.14% in category V. In the case of ENDPU, the thickest category has the highest pregnancy rate, suggesting that a thicker endometrium is considered favorable in PU measurements.

In this study, we conclude that lower endometrial thickness (mean ~ 8.5 mm) is a risk factor for successful embryo implantation. However, a thicker endometrium (mean ~ 14 mm) only beneficial at the PU time point; in other time points, an intermediate thickness (mean ~ 10-11 mm) is preferable. Moreover, the dynamic changes of the endometrium are also worth investigating, as the maximum thickness at PU time point is beneficial for successful implantation.

A high number of observational studies have assessed the association between endometrial thickness on the day of hCG triggering, in the late follicular phase, and on the day of embryo transfer. These two measurements are widely used in clinical protocols for evaluating and predicting the success and effectiveness of IVF. However, the independent contribution of endometrial advancement, maturation, and notably the transition from the proliferative phase to the secretory phase may provide insight into the optimal development pattern when measured at three consecutive important milestones: the day of hCG trigger, the day of follicular puncture, and the day of embryo transfer. The hCG trigger, imitating the endogenous LH surge in GnRH antagonist *in vitro* fertilization cycles, may lead to interindividual variation in endogenous progesterone elevation. Secretory transformation of the endometrium induced by the endogenous progesterone secretion following the hCG trigger may exhibit different patterns detectable by ultrasound measurement 35-36 hours after the trigger and consequent luteinization. However, the period between LH rise in natural cycles, hCG triggering in IVF cycles, and the subsequent progesterone rise may not exhibit standardized dynamics. Studies on natural cycles have already shown that patients with different BMI values experience varying progesterone rises following the natural LH rise (53). Only 20.6% of ovulatory women experienced a progesterone rise 48 hours following LH peak, while 69.6% had it 24 hours later, and 9.8% exhibited a progesterone rise already on the day of spontaneous LH peak. Women who had experienced a progesterone rise two days after the LH rise had a significantly higher body mass index and significantly lower serum AMH levels.

Variation in the period between hCG triggering and progesterone rise likely has implications for endometrial development between the day of trigger and the day of puncture in IVF patients. Serial ultrasound profiling during this period may help define the optimal endometrial pattern in connection with individual differences in the early luteinizing process. Describing the most optimal diagram characterizing the endometrium development in this uniquely crucial period may further support decision-making prior to embryo transfer. Individualized embryo transfer policy may include a decision-making point on the day of the puncture, whether to focus on a freeze-all strategy or fresh embryo transfer, depending not only on the absolute thickness of the endometrium (7 mm) but also on the development pattern.

### Support vector machine (SVM) model

We used the data of 537 patients with 22 properties to estimate the outcome of embryo transfer with SVM models. The average accuracy of the predictions was 61.71%. However, if we solely used the two best estimators, the prediction accuracy rose to 66.98%. We concluded that the elimination of unnecessary dimensions significantly increases the accuracy of SVM models, highlighting the importance of finding good estimators is essential for better models. In the studied dataset, age and embryo quality were the best predictors. This analysis highlighted the importance of embryo quality in the successful outcome of embryo transfer. Nevertheless, it is feasible that the individual properties of embryos could also serve as effective predictors. Age, as the second-best estimator, emphasizes the importance of age-related alterations. Interestingly, the hormone levels that change with advancing age have not been proven to be as reliable predictors as age itself. Neither FSH nor AMH levels had a significant impact on model predictions, suggesting that other age-related factors might serve as more effective estimators. In the correlation analyses, age (~0.23, ***) and embryo quality (~-0.25, ***) showed the highest correlation to unsuccessful pregnancy, suggesting that correlation analyses could be useful for identifying good estimators. However, follicle number (~-0.14, ***), FSH (~0.11, *) and AMH (~-0.11, *) levels do show some correlation with the non-pregnant category; yet, these categories do not serve as good predictors.

Factors that have no impact on model predictions might be interesting as well. For instance, sperm morphology, teratozoospermia have negligible impact on model efficacy. This can be explained by the effectiveness of whole sperm injection, where the several selection steps compensate for the poor quality of sperm cells.

The SVM models on the used medical data have been concluded to outperform previous similar models in accuracy (21). Nevertheless, it remains challenging to make a confident prediction regarding the outcome of embryo transfer. Despite the substantial improvement in predictions, opportunities for refinement still remain. The increasing amount of medical data will improve predictive models, but screening for further predictors is also required, as they could have a positive impact in ameliorating the models. Moreover, finding additional estimators could contribute to a better understanding of the role and function of biological processes during pregnancy.

## Conclusions

Our examinations indicated that AMH levels vary with age but are unaffected by BMI. In contrast, LH levels exhibit greater stability across different age groups, although they are lower in individuals with higher BMI. The interplay of age and BMI appears to have an opposing influence, as evidenced by FSH, where FSH increases with age but decreases in individuals with higher BMI. To our knowledge, there has not been any documentation of the inverse correlation between BMI and LH, FSH in the infertile female population until now. Additionally, our findings represent the first insights within the context of the Hungarian infertile female population.

Based on our results, a smaller endometrial thickness (mean ~ 8.5 mm) poses a risk for successful embryo implantation. However, a thicker endometrium (mean ~ 14 mm) is beneficial only on the day of the follicular puncture time point (PU); at the other two investigated time points, an intermediate thickness (mean ~ 10-11 mm) is preferable. Furthermore, exploring the dynamic changes in the endometrium is worthwhile, considering that the maximum thickness (ENDPU) at the time of follicular puncture time point (PU) is advantageous for successful implantation.

The effects of obesity, such as increased infertility and elevated occurrence of pregnancy complications, are naturally applicable beyond the results related to the endometrium. However, in optimally performed IVF cases, we have not observed significant differences in success rates up to clinical pregnancy.

Our SVM models on the used medical data have been concluded to outperform previous similar models in accuracy.

## Limitations

Our work has some limitations, based on a retrospective study design. Women with BMI above 30 kg/m^2^ were too few to allow analysis regarding grade I-II-III obesity separately.

In addition to the benefits of a single-investigator model, such as a controlled research environment (increased control over the study and the comparability of results), uniform methodology (minimized variables and enhanced internal validity of the study), performed by most experienced professionals, also as a limitation can be considered. The measurement errors, in the absence of a second examiner, may arise during the determination of endometrial thickness.

Another constraint is the relatively low number of patients. In certain subgroups of the study, the observed differences may not have reached statistical significance due to the small sample size.

Furthermore, we cannot assume that this sample of obese women has the same profile as women with obesity of childbearing age in Hungary, as there is no data available on women with obesity and fertility challenges who could not receive assisted reproductive technology care.

One of the main limits of this study is the lack of data regarding complications during pregnancy, as it is well demonstrated that obesity is a major risk factor for gestational complications (54).

## Data availability statement

The original contributions presented in the study are included in the article/**Supplementary Material**. Further inquiries can be directed to the corresponding authors.

## Ethics statement

This study was carried out in accordance with the Declaration of Helsinki (2000) of the World Medical Association and was approved by the Hungarian Medical Research Council (approval No. BM/18153-1/2023). All subjects have given written informed consent of the study.

## Author contributions

VV: Writing – review & editing, Writing – original draft. PB: Writing – original draft. MT: Writing – review & editing. NL: Writing – review & editing. CÉ: Writing – review & editing. KB: Writing – review & editing. ZM-C: Writing – review & editing. RS: Writing – original draft. AV: Writing – review & editing, Writing – original draft. JZ: Writing – review & editing, Writing – original draft.

## Supplementary material

Supplementary Figure 1 ROC curves of linear SVM models. Plots depict the mean ROC curves for three different datasets, employing 10-fold cross-validation. AUC values are present in the legend. ‘Full dataset’ SVM calculations encompass all 22 dimensions, ‘Selection’ represents age, total number of transferred embryos, endometrial thickness on the day of puncture, and embryo quality, ‘Best two’ comprises age and embryo quality scores.
