# Supplementary figures and images for: Endometrium development patterns and BMI groups among in vitro fertilization patients; prognostic aspects
Source: Front Endocrinol (Lausanne). 2024 Apr 26;15:1379109. doi: 10.3389/fendo.2024.1379109 (PMC11082419; doi:10.3389/fendo.2024.1379109)

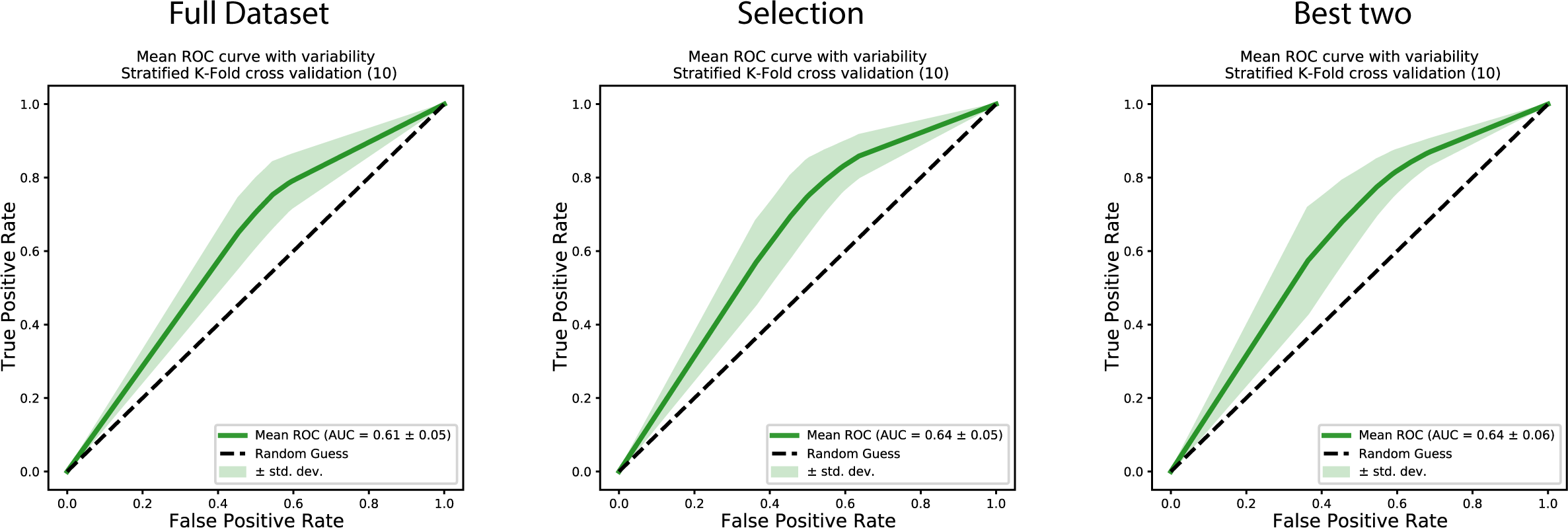

Supplement: Supplementary Figure 1 — ROC curves of linear SVM models. Plots depict the mean ROC curves for three different datasets, employing 10-fold cross-validation. AUC values are present in the legend. ‘Full dataset’ SVM calculations encompass all 22 dimensions, ‘Selection’ represents age, total number of transferred embryos, endometrial thickness on the day of puncture, and embryo quality, ‘Best two’ comprises age and embryo quality scores. [file Image_1.tif]
